# Supplementary material for: A commercial antibody to the human condensin II subunit NCAPH2 cross-reacts with a SWI/SNF complex component
Source: Wellcome Open Res. 2021 Jan 13;6:3. [Version 1] doi: 10.12688/wellcomeopenres.16482.1 (PMC7863998; doi:10.12688/wellcomeopenres.16482.1)
Supplement: Peer review history

# Peer review: Reviewer response for version 1

**DOI:** 10.21956/wellcomeopenres.18147.r42145

**Published:** 2021-02-04

**Authors:** Stephan Gruber

**Affiliations:** 1 Department of Fundamental Microbiology (DMF), Faculty of Biology and Medicine (FBM), University of Lausanne (UNIL), Lausanne, Switzerland

This important report unequivocally shows that a commercially available affinity-purified polyclonal antibody (Bethyl A302-276A) meant to target a subunit of condensin II (Cap-H2) also reacts with a component of SWI/SNF chromatin remodelling complexes. Three independent approaches with experiments performed in two research laboratories are used to reach this conclusion:

 Co-immunoprecipitation of condensin II and SWI/SNF subunits was lost when an epitope tagged-allele of the Cap-H2 was used instead of B276.

 Auxin-mediated degradation of Cap-H2 failed to eliminate enrichment of SWI/SNF subunits by B276.

 Cross-reactivity of B276 was shown by immunoblotting.

 The work indicates that a previously reported physical interaction between condensin II and SWI/SNF chromatin remodelers as well as the chromosomal colocalization may be artefacts caused by antibody cross-reactivity. More generally, the study serves as a stark reminder that the characterization of polyclonal antibodies is crucial, and it highlights the limitations of using IgG as a control reagent.

**Specific comments:**

- An in-depth discussion of the potential implications of this finding on published work would be useful.
- Typo: ‘the 276 antibody’ should read ‘the B276 antibody’

Is the work clearly and accurately presented and does it cite the current literature?

Yes

If applicable, is the statistical analysis and its interpretation appropriate?

Yes

Are all the source data underlying the results available to ensure full reproducibility?

Yes

Is the study design appropriate and is the work technically sound?

Yes

Are the conclusions drawn adequately supported by the results?

Yes

Are sufficient details of methods and analysis provided to allow replication by others?

Yes

Reviewer Expertise:

Chromosome dynamics and organization by SMC complexes.

I confirm that I have read this submission and believe that I have an appropriate level of expertise to confirm that it is of an acceptable scientific standard.

# Peer review: Reviewer response for version 1

**DOI:** 10.21956/wellcomeopenres.18147.r42148

**Published:** 2021-01-27

**Authors:** Pascal Bernard

**Affiliations:** 1 UMR 5239, CNRS - Laboratory of Biology and Modelling of the Cell, Lyon, France

In this work, Erin E. Cutts *et al.* demonstrate that a commercial antibody against NCAP-H2 (Bethyl labs A302-276A) cross-reacts with a component of a SWI/SNF complex, possibly the SMARCC1 protein, both in mouse ES and human HEK-293T cells. Experiments are well-designed, results are convincing, and methods thoroughly described. This work provides important information for the condensin and SMC community.

**Minors points**

- In Fig. 2B the authors show that an anti-Smarca4 (Swi/Snf) antibody robustly co-immunoprecipitates the SWI/SNF component Arid1a, but not NCAP-H2 when detected by the Bethyl 276 antibody. However, it is expected that this Bethyl antibody should reveal in the IPed fraction the component of the SWI/SNF complex of ~135 kDa with which it cross-reacts. Unfortunately, this information is missing.
- A comparison with the other NACP-H2 antibody from Bethyl (A302-275A) would have been valuable.
- A custom (in house) antibody has been produced that does not cross react with Swi/Snf, but no information is provided on which part of NCAPH2 has been used for immunisation.

Is the work clearly and accurately presented and does it cite the current literature?

Yes

If applicable, is the statistical analysis and its interpretation appropriate?

Yes

Are all the source data underlying the results available to ensure full reproducibility?

Yes

Is the study design appropriate and is the work technically sound?

Yes

Are the conclusions drawn adequately supported by the results?

Yes

Are sufficient details of methods and analysis provided to allow replication by others?

Partly

Reviewer Expertise:

Condensin and SMC complexes, chromatin dynamics, chromosome assembly and segregation

I confirm that I have read this submission and believe that I have an appropriate level of expertise to confirm that it is of an acceptable scientific standard.
